# A New Method Without Organic Solvent to Targeted Nanodrug for Enhanced Anticancer Efficacy

**DOI:** 10.1186/s11671-017-2174-x

**Published:** 2017-06-15

**Authors:** Shichao Wu, Xiangrui Yang, Mingyuan Zou, Zhenqing Hou, Jianghua Yan

**Affiliations:** 1 Cancer Research Center, Medical College, Xiamen University, Xiamen, 361005 China; 2 Institute of Soft Matter and Biomimetics, College of Materials, Xiamen University, Xiamen, 361005 China; 3 Department of Chemistry, College of Chemistry and Chemical Engineering, Xiamen University, Xiamen, 361005 China

**Keywords:** Green, Targeting, Anticancer, HCPT, Chitosan

## Abstract

Since the hydrophobic group is always essential to the synthesis of the drug-loaded nanoparticles, a majority of the methods rely heavily on organic solvent, which may not be completely removed and might be a potential threat to the patients. In this study, we completely “green” synthesized 10-hydroxycamptothecine (HCPT) loaded, folate (FA)-modified nanoneedles (HFNDs) for highly efficient cancer therapy with high drug loading, targeting property, and imaging capability. It should be noted that no organic solvent was used in the preparation process. In vitro cell uptake study and the in vivo distribution study showed that the HFNDs, with FA on the surface, revealed an obviously targeting property and entered the HeLa cells easier than the chitosan-HCPT nanoneedles without FA modified (NDs). The cytotoxicity tests illustrated that the HFNDs possessed better killing ability to HeLa cells than the individual drug or the NDs in the same dose, indicating its good anticancer effect. The in vivo anticancer experiment further revealed the pronounced anticancer effects and the lower side effects of the HFNDs. This new method without organic solvent will lead to a promising sustained drug delivery system for cancer diagnosis and treatment.

## Background

Over the course of the past two decades, the concentration of research was focused on improving the suboptimal pharmacokinetic properties of the chemotherapy to enhance its efficacy [1, 2]. Significant progress has been made, and many nanoparticle-based multifunctional drug delivery systems have been successfully prepared, which demonstrate a wide range of combined properties such as long circulating [3, 4], targeting [5–7], imaging [8–10], pH sensitivity [11, 12], and sustained drug release [9, 13].

**Table 1:** Experiment conditions in Fig. 3

Groups	Factors				
	HCPT/conjugate (*w/w*)	Ultrasonic power (W)	pH value	[HCPT] (μg/mL)	[Conjugate] (μg/mL)
A	1:10	200	7.0	50	500
B	10:1	200	7.0	500	50
C	1:1	200	6.5	50	50
D	1:1	50	7.0	50	50
E	1:1	500	7.0	50	50
F	1:1	200	7.0	20	20

In recent years, the non-spherical particle shape has been attracting more and more attention for their potential effect on drug delivery [14–19]. There has been already evidence that shape has an influence on many properties of the particles, such as the biodistribution and the degradation [20–22]. Most of all, the cellular internalization was proved to be strongly shape dependent [23–25]. Because the particles must be able to enter cancer cells and act on their therapeutic targets to kill them. And many studies have found that the cancer cells preferred particles with high aspect ratio [10, 26].

However, most of these methods rely heavily on organic solvents, mainly due to the hydrophobic group needed in the nanoparticle preparation processes [27]. These organic solvents may be residual within the particles and cannot be completely removed by conventional practices, such as reduced pressure distillation or freeze drying. As a result, trace amounts of organic solvents remain in the medicine, which are called residual solvents. Although residual solvents are very little and may meet the special directions published in pharmacopeias which have been strictly controlling the maximum allowable amounts of the residual solvents in pharmaceutical products, the residual solvents will be accumulating in the body and may accentuate the disease or cause other serious issues. Hence, the manufacturers have been aspiring to minimize the amount of the organic solvents used in the drug production process. Therefore, it will be a pretty major leap for the medicine, the human health, and the environment to use “green” chemistry into the pharmaceutical industry, although facing amounts of difficulties.

In this study, we developed HCPT-loaded, folate (FA)-modified nanoneedles (HFNDs) with a high aspect ratio and sharp ends via a completely green method without using any organic solvent. The pH-controlled precipitation of the HCPT and FA-modified chitosan (CS-FA) lead to nucleation of nanoneedles with nanocrystalline HCPT as the core wrapped with CS-FA as steric stabilizers. The HFNDs were found to possess good properties of targeting and imaging capability. In vitro and in vivo studies were then systematically investigated. These results highlight the great potential of FA-modified, imaging-functional nanoneedles for highly efficient chemotherapy, as well as for cancer diagnostic applications.

## Methods

### Materials

All chemicals are of analytical grade and used as received without further purification. Deionized (DI) water was used in all experiments. FA was purchased from Bio Basic Inc. 10-hydroxycamptothecine (HCPT; purity >99%) was purchased from Lishizhen Pharmaceutical Co., Ltd. Chitosan (Mw = 70 000, 90% degree of deacetylation) was obtained from Zhejiang Aoxing. *N*-Hydroxysuccinimide (NHS) and 1-(3-dimethylaminopropyl)-3-ethylcarbodiimide hydrochloride (EDC) were purchased from Sigma-Aldrich.

### Synthesis of the FA-Chitosan Conjugate

FA (10 mg), chitosan (20 mg), EDC (4 mg), and NHS (4 mg) were added into 2 mL PBS buffer solution (pH 5.5) and stirred at rt for 12 h to obtain the CS-FA suspension. Then, the suspension was dialyzed against a buffer solution (pH 10) to remove excess FA molecules. The remaining suspension was centrifuged (5000 rpm) and lyophilized for 24 h to obtain the dry CS-FA powder.

### Preparation of HFNDs

HCPT (10 μg) was dissolved in 200 μL of NaOH aqueous solution (0.1 M) to obtain solution A, and CS-FA (10 μg) was dissolved in 200 μL HCl (0.1 M) to obtain solution B. Afterwards, solution A and solution B were added dropwise into pure water (1 mL) under vigorous stirring for 30 s, and the mixture was sonicated (200 W) in an ice bath for 6 min. The suspension was centrifuged (10,000 rpm, 5 min) and lyophilized for 24 h. For the preparation of NDs, the chitosan solution was used to replace the solution B.

### Characterization

Morphology of the HFNDs was examined by SEM (UV-70) at 15 kV. The size and zeta-potential values were determined by a Malvern Zetasizer Nano-ZS machine (Malvern Instruments, Malvern). Three parallel measurements were carried out to determine the average values. Crystallinity of HFNDs was analyzed with XRD (X’pert PRO). The content of FA in HFNDs was determined by UV spectrophotometry (Beckman DU800). All samples were assayed at 281 nm. The standard curve was drawn beforehand for determining the FA concentration. The content of HCPT in HFNDs was determined by fluorescence spectrophotometry at 383 nm. The standard curve was drawn beforehand for determining the concentration of HCPT. The content and entrapment efficiency were calculated by Eqs. (1, 2, 3, and 4):

1 $$ \mathrm{Drug}\ \mathrm{loading}\ \mathrm{content}\ \mathrm{of}\ \mathrm{HCPT}\ \left(\%\right)=\left(\mathrm{weight}\ \mathrm{of}\ \mathrm{HCPT}\ \mathrm{in}\ \mathrm{HFNDs}\right)/\left(\mathrm{weight}\ \mathrm{of}\ \mathrm{HFNDs}\right)\times 100\% $$

2 $$ \mathrm{Entrapment}\ \mathrm{efficiency}\ \mathrm{of}\ \mathrm{HCPT}\left(\%\right)=\left(\mathrm{weight}\ \mathrm{of}\ \mathrm{drug}\ \mathrm{in}\ \mathrm{HFNDs}\right)/\left(\mathrm{weight}\ \mathrm{of}\ \mathrm{feeding}\ \mathrm{drug}\right)\times 100\% $$

3 $$ \mathrm{Percentage}\ \mathrm{of}\ \mathrm{F}\mathrm{A}\ \mathrm{in}\ \mathrm{the}\ \mathrm{conjugation}\ \left(\%\right)=\left(\mathrm{weight}\ \mathrm{of}\ \mathrm{FA}\ \mathrm{in}\ \mathrm{conjugation}\right)/\left(\mathrm{weight}\ \mathrm{of}\ \mathrm{conjugation}\right)\times 100\% $$

4 $$ \mathrm{Drug}\ \mathrm{loading}\ \mathrm{content}\ \mathrm{of}\ \mathrm{F}\mathrm{A}\ \left(\%\right)=\left(1-\mathrm{Drug}\ \mathrm{loading}\ \mathrm{content}\ \mathrm{of}\ \mathrm{HCPT}\right) \times \mathrm{percentage}\ \mathrm{of}\ \mathrm{F}\mathrm{A}\ \mathrm{in}\ \mathrm{the}\ \mathrm{conjugation}\times 100\% $$

### In Vitro Drug Release Study

The in vitro drug release study of HFNDs was performed using the dialysis technique. The HFNDs were dispersed in a PBS buffer solution (10 mL) and placed in a pre-swelled dialysis bag (MWCO 3500 Da). The dialysis bag was then immersed in PBS (0.1 M, 200 mL, pH 7.4) and oscillated continuously in a shaker incubator (100 rpm) at 37 °C. All samples were assayed by fluorescence spectrophotometry.

### Confocal Imaging of Cells

The confocal imaging of cells was performed using a Leica laser scanning confocal microscope. Imaging of HCPT was carried out under the 382-nm laser excitation, and the emission was collected in the range of 500–550 nm. HeLa cells were seeded and preincubated at 37 °C for 24 h (5% CO_2_) before incubated with the HFNDs for 8 h.

### Cellular Uptake Measured by Fluorescence Measurement

HeLa cells were seeded in a 24-well plate (1 × 10^6^ mL/well). The plate was then incubated at 37 °C for 24 h in a humidified atmosphere (5% CO_2_). The cells were then incubated with NDs and HFNDs at equivalent concentrations of HCPT. The drug-treated cells were incubated for 6 h at 37 °C, followed by being washed twice with PBS, and digested by trypsin (0.05%)/EDTA treatment. The suspensions were centrifuged (1000 rpm, 4 °C) for 4 min. The cell pellets were washed with PBS to remove the background fluorescence in the medium. After two cycles of washing and centrifugation, the cells were resuspended with 2 mL PBS and disrupted completely by vigorous sonication. The amount of HCPT in the sonicated mixture was analyzed by fluorescence spectroscopy (excitation at 382 nm). Blank cells in the absence of drug were measured to determine the cells auto-fluorescence level as the control.

### Cytotoxicity Assays

The cytotoxicity of HFNDs was determined by MTT assay. Briefly, an adequate number of exponential phase HeLa cells were plated in quintuplicate in a 96-well flat bottomed microplate and incubated for 24 h in the presence of drug/particles. In this study, 20 μL 3-(4,5-dimethyl-2-thiazolyl)-2,5-diphenyl-2-H-tetrazolium bromide (MTT) solution (5 mg/mL in PBS) was added in each well, and the plates were incubated at 37 °C for another 4 h. Afterwards, a volume of 150 μL dimethylsulfoxide (DMSO) was added, and the plate was agitated on a water bath chader at 37 °C for 30 min. The absorbance at 570 nm was measured using a Microplate Reader (model 680; Bio-Rad).

### Biodistribution

For in vivo fluorescence imaging, DiR was encapsulated into the NDs and HFNDs. DiR-NDs and DiR-HFNDs were intravenously administered into HeLa tumor-bearing nude mice via tail veins at an equivalent dose of DiR-HCPT per kilogram mouse body weight. At predetermined time intervals, the mice were anesthetized and imaged with the Maestro in vivo imaging system (Cambridge Research & Instrumentation, Woburn, MA, USA). After 24 h, the mice were sacrificed, and the tumor as well as the major organs (spleen, liver, kidney, lung, and heart) was excised, followed by washing the surface with 0.9% NaCl for the ex vivo imaging.

### Tumor Inhibition In Vivo

When HeLa tumor volume of the HeLa tumor-bearing mice was approximately 60 mm^3^, the mice were randomly divided into four groups and treated by intravenous injection of 0.9% NaCl, free HCPT, NDs, and HFNDs every 3 days at a dose of 80 μg HCPT per mouse. The tumor volume and the body weight were monitored every 3 days. The tumor volume was calculated by the following formula: tumor volume = 0.5 × length × width^2^.

After 21 days, the mice were sacrificed, followed by the tumors excised and weighed. Then, the tumors were fixed in 4% paraformaldehyde overnight at 4 °C, embedded into paraffin, sectioned (4 μm), stained with hematoxylin and eosin (H&E), and observed using a digital microscopy system.

### Statistical Analysis

The statistical significance of treatment outcomes was assessed using Student’s *t* test (two-tailed); *P* < 0.05 was considered statistically significant in all analyses (95% confidence level).

## Results and Discussion

### Synthesis of the FA-Chitosan Conjugate

**Fig. 1:**
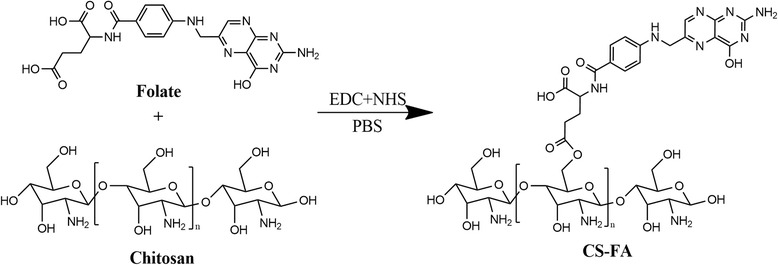
Synthetic route of CS-FA conjugate

**Fig. 2:**
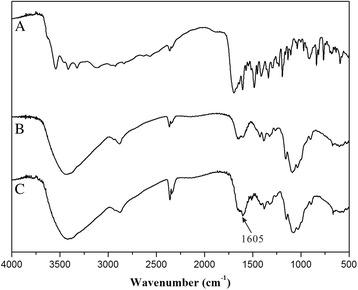
FTIR spectra of (*a*) FA, (*b*) chitosan, and (*c*) CS-FA

First, we conjugated FA to chitosan by an amidation reaction between the carboxylic end group of FA and the amidogen of chitosan (Fig. 1). The structure of the conjugation (CS-FA) was confirmed by Fourier transform infrared (FT-IR) spectroscopy. As shown in Fig. 2, the peak at 1605/cm became stronger in the IR spectrum of CS-FA than that of chitosan, corresponding to C=O stretching vibration of the new amido bond. The result indicated that FA was successfully conjugated to the amidogen of chitosan via amido bond. In order to investigate the percentage of FA in the conjugation, a standard curve was set up by ultraviolet spectrophotometry. And the percentage of FA was calculated to be 23.4 ± 2.5%.

### Preparation of HFNDs

**Fig. 3:**
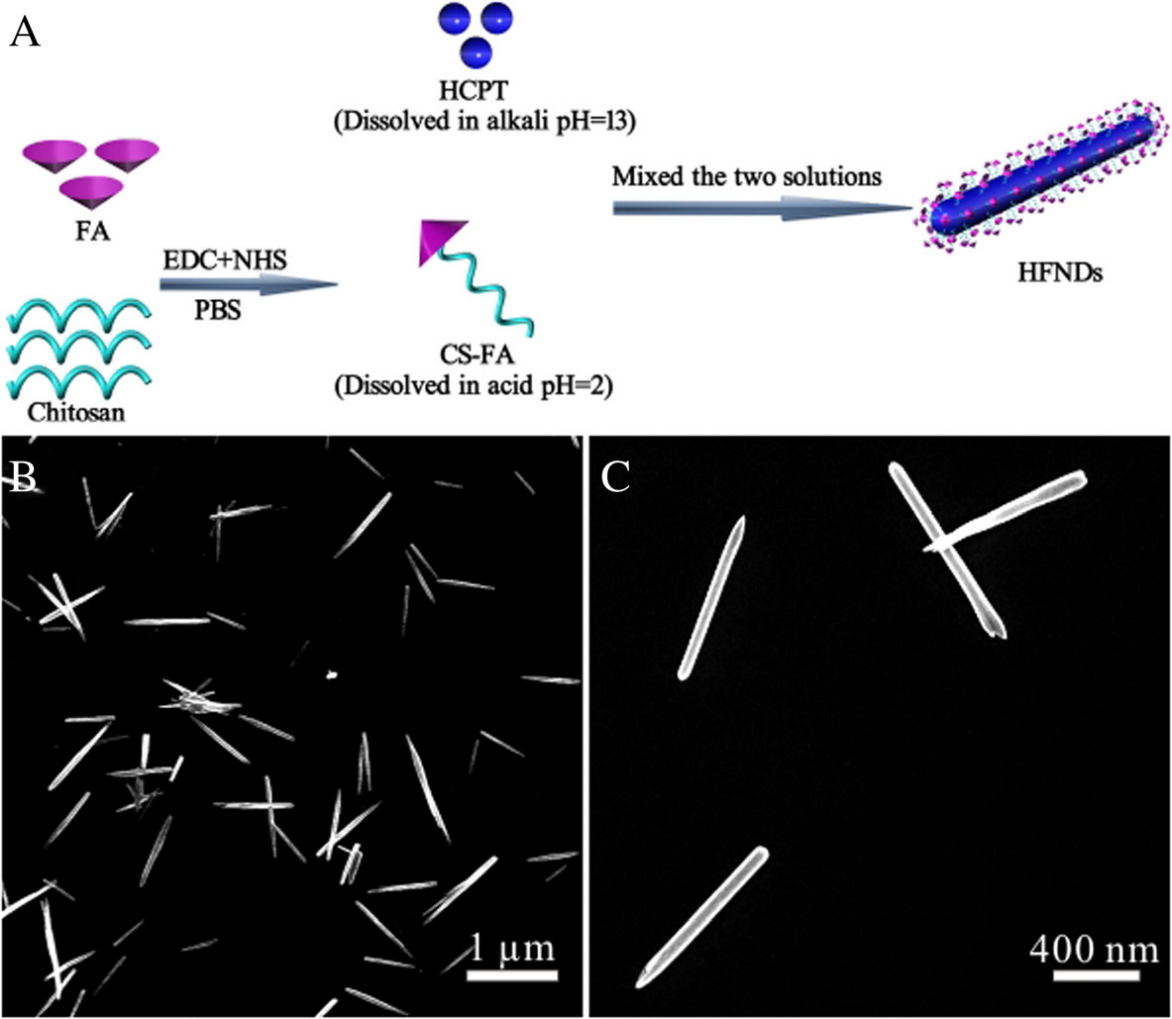
**a** Illustration of the completely green method of preparing HFNDs. **b**, **c** The SEM images of HFNDs

It is common knowledge that the solubility of HCPT in water is extremely poor, but it can dissolve in alkali. The chitosan is just the opposite: soluble in acids and insoluble in water. And the CS-FA showed the similar solubility to the chitosan. Hence, the HCPT and the CS-FA were dissolved in alkali and acids, respectively. When the two solutions were mixed, neutralization reaction would happen. The produced mixture was controlled to be neutral, which would be a poor solvent for both HCPT and CS-FA. The decrease of the solubility triggered by pH changes provided an opportunity for the nucleation of HCPT nanoneedles and the accompanying coprecipitation of CS-FA onto the growing HCPT nanoneedles (Fig. 3a). The dynamic nucleation and precipitation of the nanoneedles under ultrasound, plus the active occlusion of the soft ingredient CS-FA, led to the formation of HFNDs, instead of bulk HCPT crystal. To optimize the formulation conditions, a condition experiment was designed to study the effect of the ratio of HCPT to CS-FA, ultrasonic power, pH of the produced mixture, and concentration of the produced mixture on the morphology of HFNDs (Table 1).

**Fig. 4:**
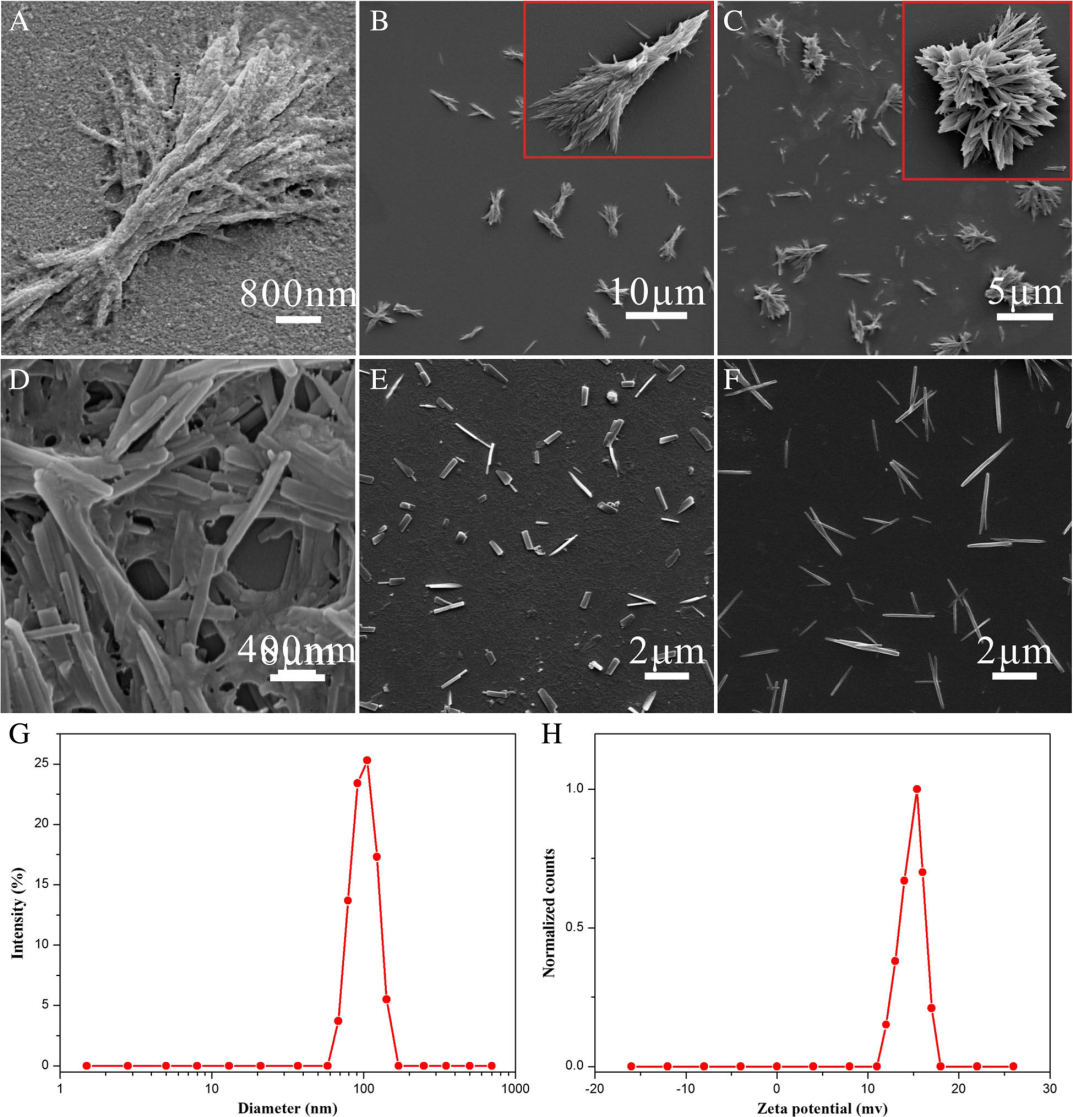
**a**–**f** The SEM images of HFNDs under different conditions (see details in Table 1). **g** Particle size distribution of the HFNDs. **h** Zeta potential of the HFNDs

Figure 4 shows the morphology of the particles under different conditions. When CS-FA was too much, excess CS-FA would attach to the surface of the produced particles (Fig. 4a). While CS-FA was too little, the CS-FA was not able to stop the growth of the HCPT nanoneedles, which were prone to aggregate (Fig. 4b). As the nucleation was triggered by pH changes, the pH of the produced mixture played an important role in the preparation process. The pH should be controlled to neutral, or the crystallization would be damaged (Fig. 4c). The ultrasonic power also had a great influence on the morphology of the HFNDs. The nanoneedles would aggregate at low power (Fig. 4d) and break down to fragments at high power (Fig. 4e). Moreover, the concentration of the produced mixture was found to have a great effect on the size of HFNDs. The size decreased by increasing the concentration (Figs. 3b and 4f).

Figure 3b, c shows the optimized needle-shaped morphology of the HFNDs with an average length of about 800 nm and the width of about 80 nm. The result of DLS measurement shows a size of 104.3 ± 5.7 nm (Fig. 4g) and a zeta potential of +16.3 ± 1.9 mv (Fig. 4h). What’s more, a 2 wt% HFNDs dispersion showed good stability for 2.5 days at least. Since there is no fluorescence signals from CS-FA, we can measure HCPT drug-loading content of the HFNDs by using the fluorescence characteristics of HCPT. The HCPT drug-loading content of the HFNDs was 70.2 ± 3.1%, and the encapsulation efficiency was 83.1%. And the FA content of the HFNDs was 7.0%, which was calculated via the percentage of FA in CS-FA.

### XRD Analysis

**Fig. 5:**
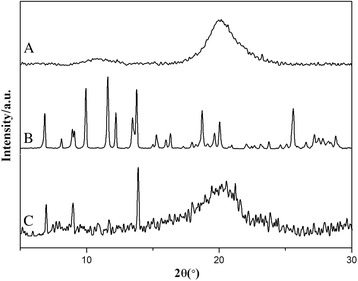
The XRD patterns of (*a*) chitosan, (*b*) HCPT, and (*c*) MHNDs

**Fig. 6:**
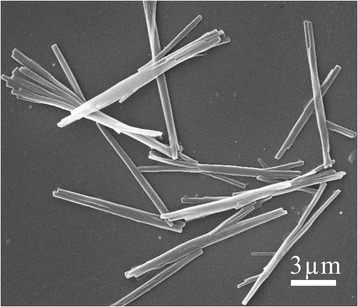
The SEM image of HCPT bulk crystals

As is well-known, the form of drug greatly affects the properties of the nanoparticles. Hence, it is of high importance to understand the form of HCPT in the HFNDs. X-ray diffraction was employed to detect the form of HCPT within the HFNDs (Fig. 5). It is clear that pure HCPT show many sharp crystalline peaks, representative of the characteristics of high crystallinity. While the broad peaks of semicrystalline chitosan still existed in the XRD pattern of the HFNDs, a majority of peaks belonged to HCPT, suggesting the high crystallinity of HCPT. In short, the XRD results suggest that HCPT is in the crystalline state in HFNDs. Moreover, the growth kinetics of HCPT within the HFNDs had been changed, mainly due to the active occlusion and confining effects of CS-FA. To investigate the effect of CS-FA in the crystallization process, the HCPT crystals were prepared without the existence of CS-FA. Figure 6 showed the morphology of the HCPT crystals. They were rod-shaped, with a length of more than 10 μm, which was totally different from that of HFNDs. This further demonstrated that the CS-FA had changed the growth kinetics of HCPT within the HFNDs.

### In Vitro Drug Release Studies

**Fig. 7:**
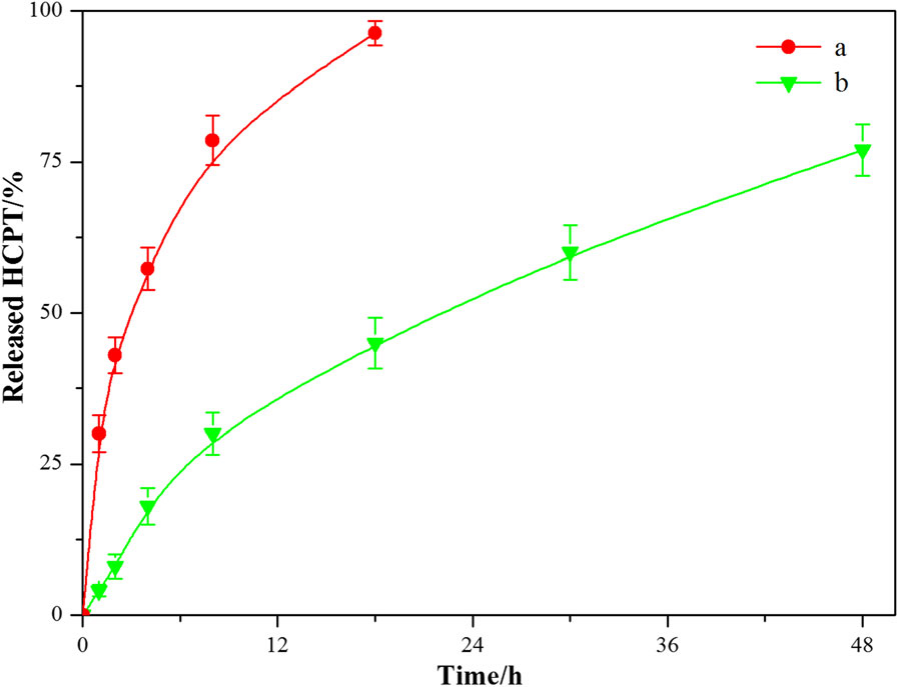
In vitro drug release profiles of the MHNDs in PBS (pH 7.4) at 37 °C. (*a*) Free HCPT; (*b*) HFNDs

Since the drug release behavior is an important property to a drug delivery system, the in vitro release studies of the HFNDs were performed using a dialysis technique, alongside with free HCPT powders. All samples were assayed by high-performance liquid chromatography (HPLC). The release profiles are shown in Fig. 7. The profile of free HCPT showed that at least 30% of the drug was released at the first sampling time of 1 h and almost 100% by 18 h. However, the release profile of the HFNDs appears to be a remarked prolonged and sustained release of the two drugs over 48 h. The prolonged drug release can be attributed to that the polymeric shell of CS-FA could limit the release of the drug in the core. These advantages could promote the application of the HFNDs for sustained drug delivery system.

### Cellular Uptake

**Fig. 8:**
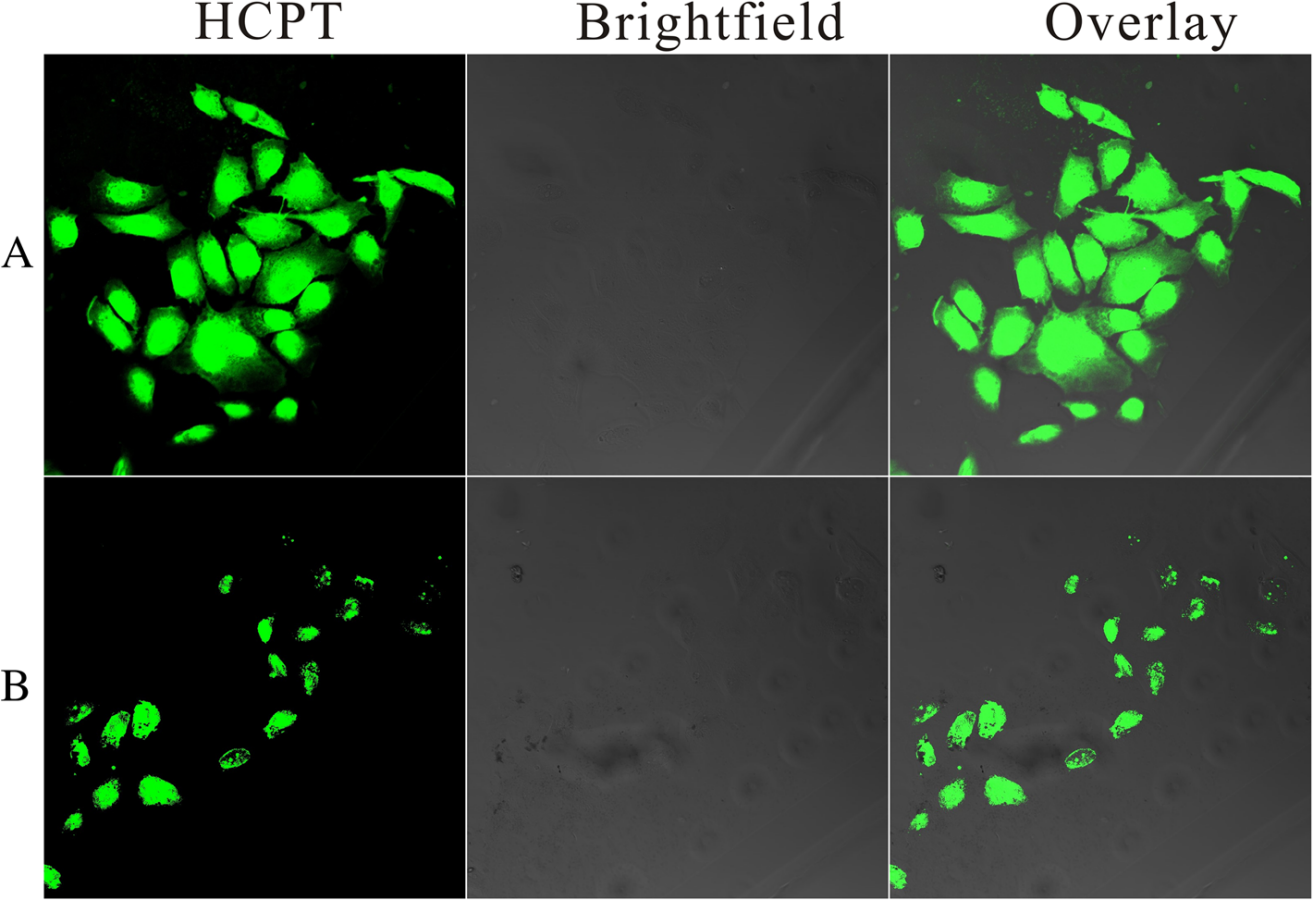
Intracellular drug delivery for 8 h at 37 °C. Confocal laser scanning microscopy images of HeLa cells incubated with **a** HFNDs and **b** NDs

No matter how the drug delivery system reaches the target tumor site, by systemic administration or by direct local administration, it is of great importance if they could penetrate within the tumor area to act on their intracellular target. Confocal laser scanning microscopy (CLSM) was performed to assess the cellular uptake of HFNDs. To evaluate their efficiency of cellular uptake by HeLa cells, the HFNDs and the HCPT-loaded nanoneedles (NDs; drug loading = 64.7%) were incubated with HeLa cells for 8 h at 37 °C (the NDs were prepared by HCPT and chitosan via the same method as the HFNDs). As shown in Fig. 8, a much more intense fluorescence emission of HCPT was detected from the cells exposed to the HFNDs than that of those exposed to NDs after 8 h of incubation, illustrating that the FA on the surface of the particles could greatly enhance the cellular uptake.

**Fig. 9:**
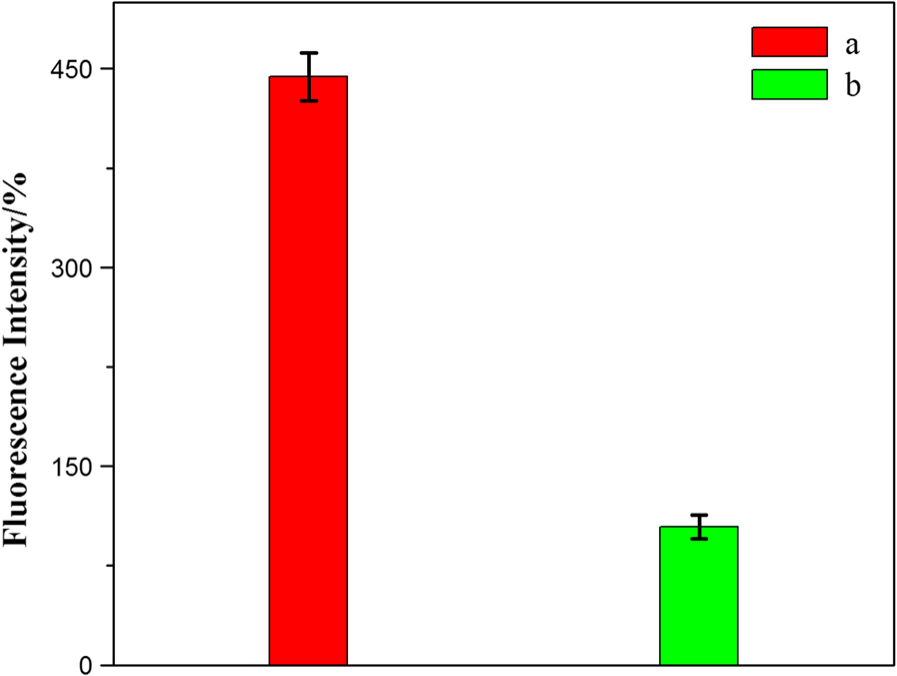
Fluorescence measurements of the HeLa cells incubated with HFNDs (*a*) and NDs (*b*) over an 8-h incubation period at 37 °C; *P* < 0.05

To further confirm that the cellular internalization rate of the HFNDs was faster, fluorescence measurements were performed to quantify the difference of fluorescence emission intensity of HCPT in the HeLa cells. Consistent with the CLSM observations, HFNDs were much more favored than NDs in the cellular internalization process (Fig. 9). This further validated the targeting property of HFNDs.

### Cytotoxicity Assays

**Fig. 10:**
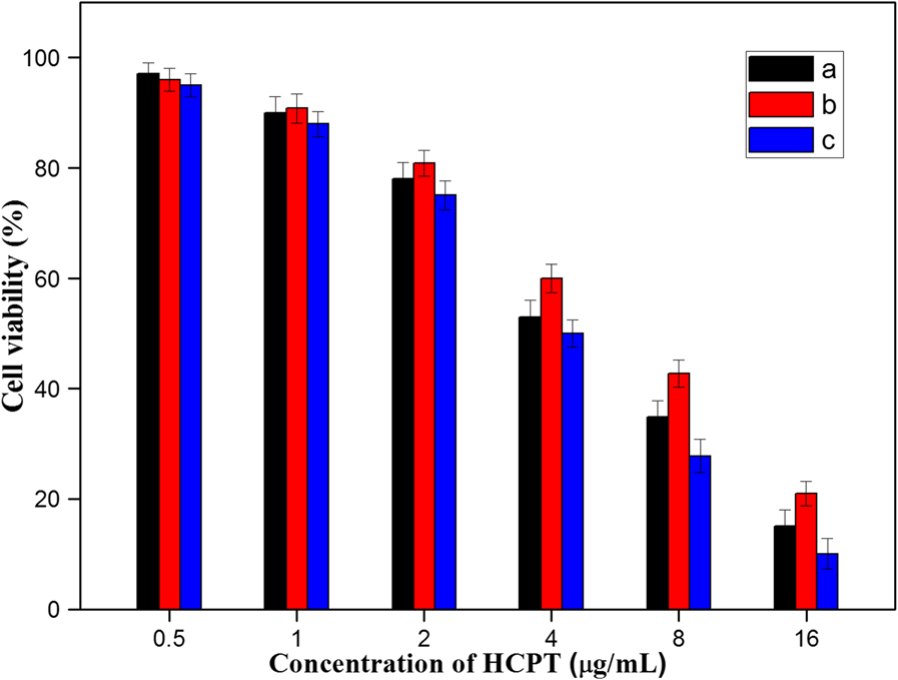
In vitro cell viability of HeLa cells treated with (*a*) free HCPT, (*b*) NDs, and (*c*) HFNDs after incubation of 24 h. *P* < 0.05

To further investigate the possibility of utilizing the HFNDs for local drug delivery, we tested the killing ability of the HFNDs to cancer cell. The cytotoxicity of HFNDs was evaluated using the MTT assay with the HeLa cells. The free HCPT and NDs containing equivalent concentrations of HCPT were used as control. The concentrations of HCPT were 0.50, 1.00, 2.00, 4.00, 8.00, and 16 μg/mL. As is shown in Fig. 10, the cytotoxicity of HCPT was higher than that of NDs, mainly because of the much faster drug release rate of HCPT than that of NDs. Nevertheless, the cytotoxicity of the HFNDs was higher than that of HCPT. This was probably due to the targeting property of FA on the surface of the HFNDs, which could help the particles to enter the cells and kill them. Thus, the HFNDs presented surprisingly good killing ability to the cancer cells. These results confirm that FA on the surface of the HFNDs can increase the cellular uptake of the particles and thus increase their killing ability to cancer cells by binding with FA receptors.

### Biodistribution

To evaluate the tumor target ability of dual-drug nanoneedles, DiR was used as a near-infrared fluorescence probe to be encapsulated into free HCPT, NDs, and HFNDs at the equivalent DiR concentration. 0.9% NaCl, DiR-NDs, and DiR-HFNDs were injected intravenously into the mice-bearing tumors derived from human cervical carcinoma HeLa cells, and their in vivo biodistributions were investigated.

**Fig. 11:**
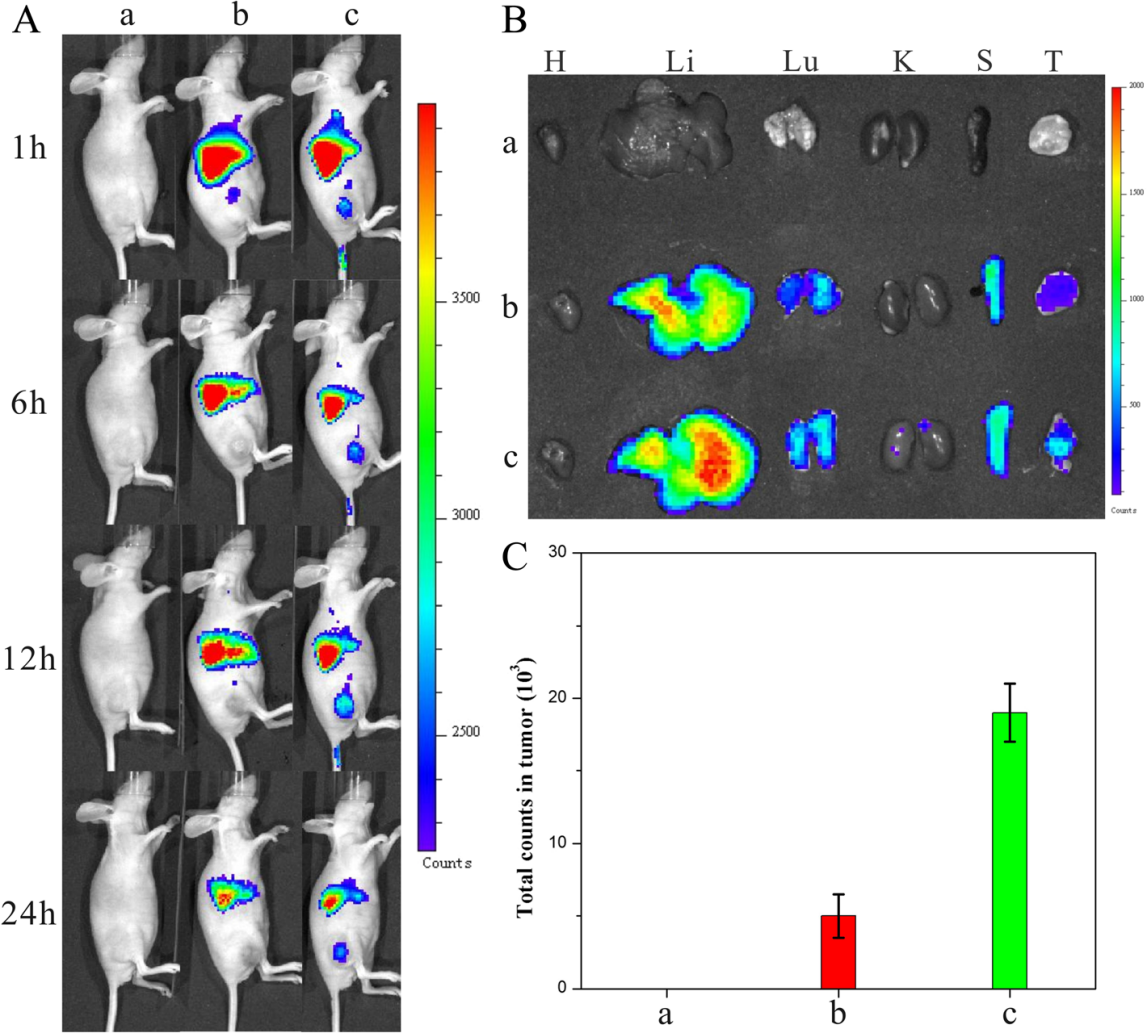
**a** Distribution and tumor accumulation of DiR-nanoparticles in HeLa tumor-bearing mice receiving intravenous injection of the indicated formulations. **b** Ex vivo fluorescence imaging of the tumor and normal tissues harvested from the euthanized HeLa tumor-bearing nude mice. The images were taken 24 h after the injection. H, Li, Lu, K, S, and T represent the heart, liver, lung, kidney, spleen, and tumor, respectively. **c** DiR fluorescence intensity in tumor tissues collected at 24 h following systemic injection. *P* < 0.05. (*a*) 0.9% NaCl, (*b*) DiR-NDs, and (*c*) DiR-HFNDs

As depicted in Fig. 11a, while no fluorescent signals were detected at tumor sites in the group of DiR-NDs, a strong fluorescent signal was visualized in the DiR-HFND group. When the total fluorescence counts were reduced all the time, the intensity of the signal at the tumor site was enhanced from 1 to 12 h, indicating that the HFNDs were accumulating in tumors during this time. After 24 h, the mice were sacrificed and the tumor tissues as well as the normal tissues were isolated for ex vivo imaging and analysis (Fig. 11b, c).The fluorescence intensity in the tumor tissue of DiR-HFNDs-treated mice was significantly higher than the other mice. It was validated that the introduction of FA offered the nanoneedles an excellent tumor targeting efficacy, leading to a higher highly efficient cancer treatment.

### Tumor Inhibition In Vivo

**Fig. 12:**
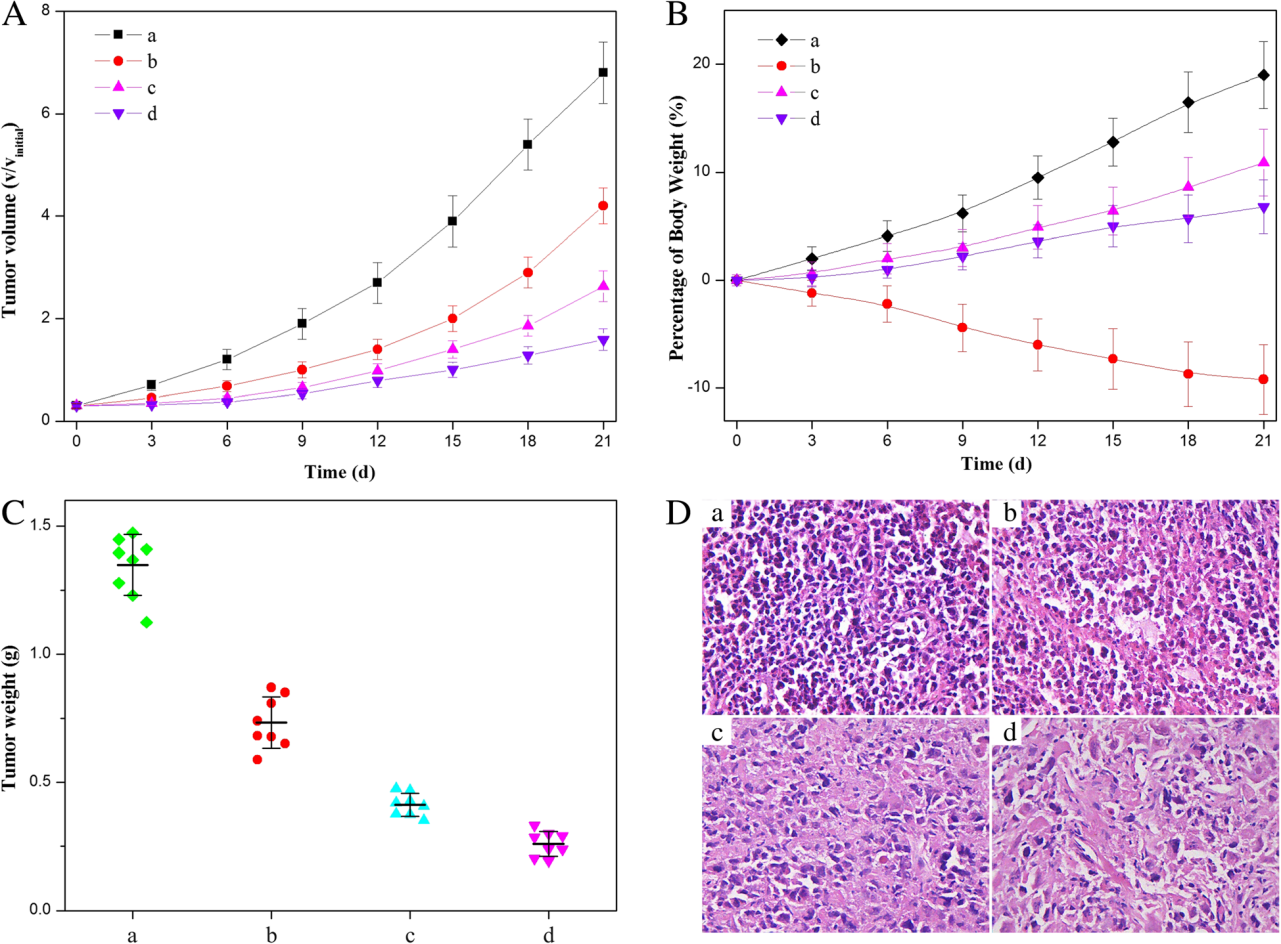
Anticancer effects of different (nano)formulations. **a** Volume change of tumor in mice during the treatment. **b** Weight change of the tumor-bearing mice during the treatment. **c** Weights of HeLa tumors after being treated by different formulations. **d** Histological section of the tumor of the mice after the treatment. (*a*) 0.9% NaCl aqueous solution, (*b*) free HCPT, (*c*) NDs, and (*d*) HFNDs. All formulations used the same concentration of HCPT in mice-bearing HeLa tumor. *P* < 0.05

To evaluate the in vivo antitumor effects, we generated HeLa tumor xenografts in Kunming mice and assessed tumor growth following the intravenous administration of 0.9% NaCl, free HCPT, NDs, and HFNDs with the same concentration of HCPT. Compared to the mice treated with 0.9% NaCl as control, the growth rate of the tumors in mice receiving free HCPT or NDs decreased gradually (Fig. 12a), indicating the significantly effective tumor growth inhibition. Of note, the HFNDs led to the most pronounced inhibition of tumor growth. At the end of experiment, the tumors were excised and weighed. As shown in Fig. 12c, it was found that the dual-drug nanoneedles had superior therapeutic efficacy compared with the other groups (*P* < 0.05). An additional evidence of the enhanced anticancer effect of the dual-drug nanoneedles was shown in the histologic images (Fig. 12d). For any drug delivery systems, the systemic toxicity that is usually encountered in the free HCPT-mediated treatment should be considered to ensure safety and effectiveness. In this work, the administration of the free HCPT resulted in the listlessness/laziness and severe body weight loss of mice (Fig. 12b), indicative of the undesirable side effects of chemotherapy. On the contrary, no obvious side effects were shown in the mice treated with NDs and HFNDs. Overall, it was indicated that the HFNDs with the superior anticancer effects as well as lower toxicity would greatly improve the efficacy of quality of life therapy.

## Conclusions

The study herein presents a completely green approach to obtain FA-modified, HCPT-loaded nanoneedles for the highly efficient chemotherapy with high drug loading, targeting property, and imaging capability. The drug release profile revealed that the HFNDs showed a sustained and prolonged release. The CLSM demonstrated the more effective cellular internalization of HFNDs than NDs. The MTT experiment indicated that the HFNDs not only showed a much higher cytotoxicity than the individual drugs and NDs. This illustrated the good targeting property of the HFNDs. This work opens a door to design new dosages of nanoparticles with completely green method, which might have a powerful effect on environmental protection in the future.
